# Obstructive sleep apnoea – diagnosis is all very well, but treatment is necessary

**DOI:** 10.7196/AJTCCM.2019.v25i2.013

**Published:** 2019-07-31

**Authors:** Richard Raine

**Affiliations:** Division of Medicine, Department of Pulmonology, Groote Schuur Hospital and University of Cape Town, South Africa

## Editorial

The article in this issue by Ouedraogo *et al*. ^[1]^ from Burkina Faso complements one from Nigeria^[2]^ in demonstrating a high prevalence of symptoms of obstructive sleep apnoea (OSA) in Africa. The Burkina Faso cohort was made up of caregivers in the outpatient department. The mean age was 31.8 years, and 9.6% of those enrolled had symptoms of OSA. The Nigerian cohort was made up of patients presenting to a medical outpatient clinic. These patients were older (mean age 53.8 years) than those in Burkina Faso, and 73.1% had symptoms of sleep-disordered breathing. Both studies relied on questionnaires for diagnosis of sleep-disordered breathing, as no equipment for objective diagnosis was available.

These data suggest that undiagnosed and untreated OSA is very common in Africa, particularly in those with medical problems such as hypertension. Both articles make a plea for greater availability of diagnostic equipment to permit the accurate diagnosis of sleepdisordered breathing. Polygraph recording equipment capable of performing a level III study is relatively cheap compared with formal polysomnography, and is adequate for the diagnosis of OSA.^[3]^

The problem is greater than this, however. It is all very well to make a diagnosis of OSA, but a greater problem is how to treat it. Generally accepted treatment options for OSA include avoidance of sedative drugs and alcohol, weight loss, use of an oral appliance, continuous positive airway pressure (CPAP) and occasionally, surgery. Surgery is generally unsuccessful, and is warranted only in very carefully selected cases, with the exception of tonsillectomy, which is often very helpful if enlarged tonsils are present.

Weight loss is available to all, but unfortunately, the impact of weight loss in the treatment of OSA is not large. Studies showing weight reductions due to medical management of between 5 and 20 kg are associated with a fall in apnoea-hypopnoea index (AHI) of ~50 - 60%. Bariatric surgery has shown greater weight loss and reduction in AHI; however, very few patients achieve complete cure.^[4]^ Weight loss is an essential component of the management of OSA, often as an adjunct to other forms of therapy, and has desirable effects on other comorbidities such as diabetes mellitus and hypertension.

CPAP therapy administered by a nasal or oronasal mask has become the standard accepted treatment for OSA. A commonly asked question is whether CPAP is beneficial. There is good evidence to suggest that the use of CPAP in many patients with OSA significantly reduces daytime sleepiness, with consequent improvement in cognitive function and quality of life. This improvement is particularly seen with CPAP adherence ≥4 hours/night.^[5]^ CPAP was disappointingly ineffective in reducing cardiovascular events in a group of non-sleepy patients with OSA and incident cardiovascular disease, although improvements in quality of life were present.^[6]^ It should be noted, however, that CPAP adherence in this study was a mean of 3.3 hours/night. A recently published study from Finland^[7]^ has shown that using CPAP for 6.4 hours/night was associated with a 36% reduction in cardiovascular events compared with a control group who had discontinued CPAP use. Recent data from a long-term observational cohort in the Sleep Heart Health Study^[8]^ has shown a reduction in all-cause mortality (hazard ratio 0.58, range 0.35 - 0.96) in CPAP-treated patients with OSA. This difference appeared after 6 - 7 years of CPAP therapy. CPAP is therefore effective therapy for OSA, but needs careful attention to adherence, as longer periods of use are associated with less sleepiness and better medical outcomes. The cost of CPAP, however, remains a major problem in limiting effective treatment of OSA in poorly resourced areas.

A number of oral appliances are available, with the most effective requiring fitting and customisation. Oral appliances are generally effective in milder disease and, being better tolerated, adherence to use is good. Snoring is often reduced, but the effect on sleepiness is variable. Although the cost of an oral appliance is generally about half that of CPAP, their less predictable and lower efficacy in more severe disease makes such appliances less useful for general use in OSA.^[9]^

Overall, treatment of OSA remains a major barrier to management of sleep-disordered breathing in financially constrained settings. Diagnosis without treatment is unsatisfactory, and therefore resources for both need to be available for effective management of this common problem. Weight loss remains desirable for effects on other comorbidities, even though its effectiveness in treating OSA is not large.
